# More care out of hospital? A qualitative exploration of the factors influencing the development of the district nursing workforce in England

**DOI:** 10.1177/1355819618769082

**Published:** 2018-05-12

**Authors:** Vari M Drennan

**Affiliations:** Professor of Health Care & Policy Research, Centre for Health & Social Care Research, Faculty of Health, Social Care & Education, Kingston University and St George’s University of London, UK

**Keywords:** district nursing, home healthcare, qualitative methods, workforce

## Abstract

**Objectives:**

Many countries seek to improve care for people with chronic conditions and increase delivery of care outside of hospitals, including in the home. Despite these policy objectives in the United Kingdom, the home visiting nursing service workforce, known as district nursing, is declining. This study aimed to investigate the factors influencing the development of district nursing workforces in a metropolitan area of England.

**Methods:**

A qualitative study in a metropolitan area of three million residents in diverse socio-economic communities using semi-structured interviews with a purposive sample of senior nurses in provider and commissioning organizations. Thematic analysis was framed by theories of workforce development.

**Findings:** All participants reported that the context for the district nursing service was one of major reorganizations in the face of wider National Health Service changes and financial pressures. The analysis identified five themes that can be seen to impact the ways in which the district nursing workforce was developed. These were: the challenge of recruitment and retention, a changing case-mix of patients and the requirement for different clinical skills, the growth of specialist home visiting nursing services and its impact on generalist nursing, the capacity of the district nursing service to meet growing demand, and the influence of the short-term service commissioning process on the need for long-term workforce development.

**Conclusion:**

There is an apparent paradox between health policies which promote more care within and closer to home and the reported decline in district nursing services. Using the lens of workforce development theory, an explanatory framework was offered with factors such as the nature of the nursing labour market, human resource practices, career advancement opportunities as well as the contractual context and the economic environment.

## Introduction

Many health care systems are increasing ambulatory and primary care services to address population changes and contain rising health care costs.^1^ One element is the delivery of nursing within the home, known variously as home health care, home visiting, public health, community or district nursing. Home visiting nursing services feature in many, but not all, health care systems. Some countries such as the United Kingdom (UK) and United States of America (USA) developed these in the 19th century,^2^ while others introduced them more recently, for example Japan^3^ and China.^4^ Home visiting nurses represent a small percentage of the nursing workforce, with figures ranging from under 7% of registered nurses (RN) in Australia (2015),^5^ about 9% in the National Health Service (NHS) in England (2017)^6^ to 13% of employed RNs in the USA (2016).^7^ In the UK, home visiting nurses, commonly referred to as district nurses provide services to housebound, mainly older people with long-term conditions or those who are terminally ill. In England, recent moves to increase self-management among people with long-term conditions, the provision of palliative care at home, along with a desire to reduce unplanned hospital admissions^8^ point to the need for more and differently skilled district nursing. Yet, recent reports have highlighted declining numbers and low morale among the district nursing workforce.^9,10^ Thus, between 2009 and 2017, the district nursing workforce fell by 14%, from 32,699 full time equivalents in September 2009 to 28,237 in July 2017 (including RNs with and without district nurse qualifications).^6^ These developments appear to run counter to the policy aims of enhancing care in the community.

Developments in the district nursing workforce have to be interpreted in the wider context of workforce development. Workforce development is theorized to encapsulate more than just employment training in, but to also include employer engagement with the labour market, integrative human resources practices and career advancement opportunities,^11^ all of which is shaped by the context and economic environment.^12^ At the level of individual organizations, workforce development includes improving performance through providing learning opportunities as well as responsiveness to changes that affect workforce effectiveness.^13^ There is some evidence that has examined the home visiting nursing workforce in relation to current and required nurse numbers (see for example from the UK,^14^ Australia,^15^ and the USA^16^) but there is a lack of further exploration of the factors that influence the broader aspects of workforce development with respect to the district nursing workforce in the UK or in other countries.

In this study, the focus is on the NHS in England. It has a strong suite of human resource practices guided by the NHS Constitution, which sets out the rights for patients, public and staff in the NHS, and nationally agreed employment terms and conditions.^17^ District nursing services are commissioned by local Clinical Commissioning Groups (CCGs), which are responsible for the planning and commissioning of health care services for their local area, mainly through block contracts which involve payment for a broadly defined set of services. Senior NHS nurses in provider and commissioning organizations are required to participate in regional workforce development planning, led by Health Education England (HEE), the national body responsible for coordinating education and training within the health and public health workforce in England. This study investigated the factors influencing workforce development of the district nursing service in English metropolitan areas from the perspectives of senior nurses in provider and commissioner organizations.

## Methods

The methodology drew on the interpretivist tradition and data were collected through semi-structured telephone interviews in 2014.^18^ A purposive sample was identified of senior nurses in 8 organizations providing district nursing services in the 12 CCGs in the metropolitan area of South London (resident population of three million living in inner city and suburban areas). Invitations to participate were sent by publically available NHS email addresses. A topic guide was developed with the study advisory group of NHS managers and academics. Topic areas included views on: strengths and weaknesses in the current district nursing workforce, factors supporting or inhibiting development, as well as the perceived direction workforce development should take. Participant verification was used within the interview to confirm the researcher’s understanding and interpretation.^18^ Interviews were conducted face-to-face or by telephone, as preferred, by the author and were of 25 and 50 min duration. With permission, interviews were recorded or notes were taken and transcribed with identifying features removed. Participants provided written and verbal consent. The data were thematically analysed and through an iterative analytical process, tested in subsequent interviews. By the final interviews, no different views were offered. The analysis and interpretation were then further tested for credibility and confirmed in a seminar with a different group of 30 senior community nurse managers and educators, from across Greater London and the surrounding area. Invitations were sent using publically available information on NHS organizations’ websites. The study received ethical approval from Kingston University Research Ethics Sub Committee for the Faculty of Health, Social Care & Education in November 2013.

## Findings

Interviews were undertaken with six senior nurses in provider organizations and with eight CCG senior nurses (total interviews =14). All participants had 10 or more years’ experience in senior positions and all but one were female. All were or had been involved to some extent in HEE processes for the allocation of funding for nursing workforce development.

The district nursing service (known as adult community nursing in some) included in this study only provided services to housebound adults. All district nursing services were reported to include different grades of registered nurses as well as health care assistants. Most participants reported that their district nursing services had been recently reorganized for one of the following reasons:

- As a result of merger with, or separation from, other NHS funded organizations (a requirement with the enactment of the Health & Social Care Act 2012^17^),
- Through the creation of new multidisciplinary or specialist teams,
- To align with other services (such as general practice) as required by commissioners.

The wider organization and commissioning context were identified as a significant influence on district workforce development. The analysis of the interview data identified five interlinked themes: (i) staffing the service: the challenge of recruitment and retention; (ii) changing case-mix of patients and the requirement for clinical skills; (iii) specialist versus generalist nursing services; (iv) capacity of the district nursing service; and (v) influence of the service commissioning process. We address each of these themes in turn.

### Staffing the service: The challenge of recruitment and retention

Participants from provider services described vacancy levels that were constant and problematic. They explained the challenges faced in recruiting the right calibre of registered nurses and retaining them. Some described a constant need for recruitment of junior registered nurses who tended to stay for relatively short periods of time:

*It’s just an endless struggle to keep these posts filled*. (Provider nurse 6)

All participants described negative consequences of high staff turnover and high use of agency staff, including loss of continuity of care for patients:

*It means you are always sending someone new to some patients.* (Provider nurse 3)

High staff turnover was also seen to have resulted in loss of team and multidisciplinary relationships, which were noted as important to the quality and safety of care of patients living at home. A vicious circle of impact within teams was described:

*It’s a downward spiral where vacancies lead to more stress for the rest of the team, so more sickness, so more vacancies needing cover by agency staff.* (Provider nurse 10)

Issues related to salary and its consequences for recruitment and retention were frequently discussed. Participants who managed services in areas where the nationally agreed level of payment supplements for NHS staff was lower described how nurses they had recruited often quickly left to work in adjacent areas which paid the higher rate. Some participants pointed out that although the area they were working in was a metropolitan area, patients were often widely dispersed. This meant that the service required nurses who are able to drive a car, and who preferably were car owners and willing to use them for work. The consequences of employing staff who did not drive (‘walkers’) were described as problematic as ‘they slow us down’ (provider nurse 11). This was raised as an issue for recruiting to any district nursing post (compared to recruiting for a hospital-based position), but particularly for lower paid grades:

*When they get down to the brass tacks of the changing the insurance and mileage *[reimbursement]*, which is now less favourable, it can make them* [job applicants] *change their minds about working for you*. (Provider nurse 3)

All participants were very aware of the demographic profile of the district nursing workforce as an ageing one, *‘Shall we say* [the staff are] *on the mature side*’ (provider nurse 4), which needed appropriate workforce planning and training to replace, *‘it’s a demographic time bomb’* (provider nurse 6). They commented on the importance of having *‘good leaders in district nursing teams’* (provider nurse 11). Not all organizations required their team leaders to have a district nursing qualification. Opinions were divided on the value of this qualification, which is known as specialist practice (district nursing) qualification. There were those who firmly believed that the job could not be done without it:

*It* [the district nurse qualification] *makes such a difference as to how they approach the job, the patients, the staff*. (Provider nurse 8)

There were others who suggested that team leaders needed to learn about caseload and people management in the out-of-hospital setting but that this did not require a 12-month university course. There was, however, consensus that there needed to be a clearly described career pathway into and through district nursing, which was currently absent. Linked to this view was a consensus among the senior nurses that ongoing reductions in NHS regional funding to individual provider organizations for continuing professional development for their nursing workforce had impacted negatively on their attractiveness to potential employees and their ability to retain nursing as well as their plans for changing and improving skills and performance.

### The changing patient case-mix and the requirement for clinical skills

All participants from provider organizations stated that the patient case-mix was changing, thus creating increased demand on the district nursing service. There was a perception of higher volumes of patients who needed complex, technical procedures to be carried out in their homes compared to *‘say five or six years ago’* (provider nurse 3). There was also a higher number of people choosing to die at home, and this changing patient mix was seen to require more time from staff than those needing simple procedures and *‘often needed two staff rather than one’* (provider nurse 8). In workforce terms, providers and some commissioner participants commented on the need to have nurses with advanced technical and clinical skills to respond to this growing demand. A small number of provider organization participants pointed to the challenge for nurses to maintain the confidence and competence in specific technical skills especially where cases in which these skills were required were relatively uncommon:

*The last time that team had one* [patient with a recent tracheostomy] *was seven years ago*. (Provider nurse 3)

Some participants pointed to previous workforce development initiatives such as staffing rotations between community nursing and hospital services as a possible mechanism for both maintaining clinical skills and also giving a wider cadre of nurses an opportunity for gaining clinical experience in home settings. When asked why these schemes no longer existed, participants suggested that changes in financing, managers and hospital shift patterns had all contributed.

Two participants suggested that lack of clinical competency in rarely seen conditions along with technical procedures were used by some district nurses to draw boundaries around their caseload. This was seen to be one of the few ways that district nurses had to control patient numbers on their caseloads. For other participants, this was seen to reflect a wider change in district nurse patient case mix as a consequence of a growth in specialist services provided in people’s homes.

### Specialist versus generalist nursing services

Participants reported that there were increasing numbers of specialist teams or specialist nurses being commissioned to provide services to people in their own homes, which they contrasted with the generalist district nursing service. Some participants expressed concern for the continuity of care for patients where the specialist teams only looked after housebound patients only for a specific time period (e.g. post hospital discharge) or for a single condition (e.g. diabetes when patients had multiple comorbidities). Others could see the value in specific teams or nurses with specialist expertise for some conditions or at critical periods. However, most expressed concerns about the consequences this changing balance between specialist and generalist nursing services had for the district nursing service and workforce development as a whole. Some participants considered that continuing growth of specialist services would leave the district nursing service only undertaking work that was unattractive, for whatever reason, to others: *‘All the patients, or work, no one else wants’* (commissioner nurse 9).

At the extreme, participants perceived the growth of specialist teams and the reduction of the sphere of work for generalist nurses as likely to make the district nursing work very unattractive and would therefore further increase ongoing challenges of recruitment and retention of staff. They also considered that such a trend towards specialist teams would make it harder for generalist district nurses to maintain advanced clinical skills and therefore map out attractive career pathways in the community.

### Capacity of the district nursing service

After noting the changing profile of the patients, nearly all participants from provider services commented on increased levels of patient contacts, higher levels of staff activity and ‘busy-ness’ of district nursing teams. There appeared to be consensus that this ‘busy-ness’ led to the nurses becoming *‘task focused’* (commissioner nurse 12). This was viewed as a problem by participants from commissioning organizations who were looking for district nursing services to actively engage in the broader agenda of increased anticipatory care for people with long-term conditions to prevent unplanned hospital admissions:

*They do the dressing and go* [leave the patient’s home], *rather than make every contact count in terms of promoting self-management, health promotion and anticipating and addressing problems immediatel*y. (Commissioner nurse 12)

Commissioner nurses considered that nursing teams could use their staff resources more efficiently. Some pointed to the lack of patient acuity or dependency tools to understand the resource demand and manage staff allocation:

*They* [the district nursing services] *don’t seem to have any way of categorising the patients in terms of the illness or dependency on the service. I don’t see how they can understand the demand and allocate staff accordingly*. (Commissioner nurse 1)

In contrast, participants from provider organizations reported on burdensome administrative or infrastructure issues, internal to their organization or externally imposed, which were seen to increase the demands on time and to reduce overall efficiency. Examples were given of increased paperwork to be completed in order to qualify for payment of added care responsibilities. Some participants flagged that inefficiencies resulted from under investment in information technology to district nursing and this was seen to be particularly challenging where community services formed only a small part of an acute hospital organization:

*So we‘ve now got more computers that the nurses can use – but still not mobile* [information technology (IT) for patient records] a*nd if there is a problem the IT support from* [name of hospital] *puts us* [the district nursing service] *at the bottom of the priority list after all the acute services*. (Provider nurse 4)

These types of issues led some participants to question whether the district nursing service workforce had to include more *business workforce support* (commissioner nurse 5) in the future to become more efficient. It also raised questions about the extent to which team leaders and senior nurses in district nursing were involved in the planned development of staff when their focus was on patient delivery.

### The influence of the commissioning process

Divisions were evident in the views between those from commissioning and those from provider organizations when discussing the commissioning process. As noted earlier, district nursing services in the NHS are commissioned by block contracts and some commissioner participants commented that this approach would not provide *‘enough granularity’* (commissioner nurse 13) to understand the activity and outcomes of district nursing services. They suggested that CCGs were *‘paying for over-performance’* (commissioner nurse 14) which did not address the pressing issue of improving the care of people with long-term conditions and reduce hospital use. Conversely, some provider participants suggested that CCGs preferred block contracts because these contracts would mask the level of their activity and ensured that the contract price did not increase:

*It* [block contracts] *keeps their costs down but not ours*. (Provider nurse 8)

Interviews revealed that some areas experienced quite adversarial relationships between commissioners and provider organizations with regard to the problems and costs of the district nursing service, while others described more collaborative relationships to address workforce development. Many of the participants discussed uncertainty in continuation of contracts for district nursing services, namely the prospect of contracts being removed from current provider organizations. This was seen to have consequences for workforce development:

*So there is a sense of ‘short-term-ism’ in contracts which makes it very difficult to plan long term for a workforce*. (Provider nurse 10)

Examples were given of integration initiatives with local authority funded social care teams or general practice. These were, however, not sustained in subsequent commissioning rounds due to changes in commissioners or reduction in funding available. Such developments were cited as examples that made long-term planning workforce development challenging. Most participants agreed that these concerns were not new, however, but rather reflected persistent, long standing and enduring problems.

## Discussion and conclusion

This qualitative study examined the factors that influence workforce development of the district nursing service in the English NHS from the perspectives of senior nurses in provider and commissioner organizations in South London. It identified a range of factors many of which mainly hinder rather than supporting the development of the district nursing workforce. From the perspective of provider organizations, these factors included difficulties in being able to recruit and retain a sufficient number of nurses. It also included changing and increasing demand for the service, which, while offering the potential for growth and career development, was viewed negatively in the context of the local contracting process for district nursing, which was considered to inhibit any such developments. There were some examples of workforce development collaboration between commissioners and provider services, but this did not appear to be the case everywhere. The data highlighted the impact of the wider (local) system on workforce development. Key factors included uncertainty created by short-term service contracts, disruption caused by short-term reorganizations of service and team configurations, along with competition from an increasing number of specialist home visiting services, which was seen to fragment the work and demand for generalist home nursing services. These were perceived to inhibit workforce development even when the relationships were more collaborative. In addition, the national NHS system level funding support for employer defined continuing education and clinical careers in district nursing were observed to be diminishing.

Theories of workforce development suggest it as a dynamic system of influences internal and external to an organization,^13,14^ which include the wider labour market, human resources practices, career advancement opportunities, and the wider context and economic environment.^11^ With regard to the wider labour market, district nursing or home visiting nursing represents only a small group in the overall nursing labour market as noted earlier.^5,6,17^ An increased demand for nurses from all sectors and concomitant shortage in supply^20,21^ means that employers are competing for nurses from the same diminishing labour pool. Participants in this study described significant difficulties in recruiting and retaining nurses, reflecting experiences elsewhere in England^22^ and suggesting that this is not an isolated phenomenon. Factors such as lower financial incentives for district nursing compared to other nursing work have been identified here which worked as ‘push’ factors for nurses to join a different part of nursing labour market. Evidence on factors influencing the decision of nurses to work as home visiting or district nurses remains scant but studies that do exist suggest that the intention to remain is linked to factors such as perceived reasonable workload along with adequate pay and benefits.^23^

This study highlights the importance of rising demand for home nursing and the growing number of more complex patients. There is evidence of increasing referrals to district nursing service in England^24^ and similar developments have been reported from South Australia.^25^ The reported increase in the complexity of patients in this study was matched by the reported growth in the use of specialist teams and nurses in the community. This was seen to have created challenges for workforce development in that senior nurses were concerned with maintaining capacity to respond to these changing needs while balancing this against the possibility that these patients would not be referred to the generalist district nursing service. Such challenges risk the further fragmentation of nursing work, which was also seen to challenge the creation of attractive work and career development opportunities for those in district nursing services. Nurse concerns about increased specialization at the cost of generalist community nursing services have been noted before,^26^ while this study reports for the first time the ambiguities these issues pose for nurse managers in addressing workforce development.

With regard to career advancement opportunities, study participants were unanimous in arguing for creating attractive career pathways for nurses, but they were divided in their views about the nature of the education and training required for district nursing. The curriculum and length of educational preparation for community nursing differ between countries and there is no comparative research evidence available on effectiveness of models. This is an area worthy of further investigation as is the impact of organisational funding (or lack of it ) for continuing education.

Participants pointed to the challenges posed by a perceived repeat disruption created by reorganizations of structures and teams in relation to sustainable workforce development. Evidence from elsewhere suggests that reorganizations of health services can take at least three years for the service to return to earlier levels of functioning.^27^ The senior nurses participating in this study had divergent views of the routes and endpoints to the development of the district nursing workforce. This divergence is reflected in the wider narrative around the district nursing workforce that works away from the public gaze with a low-status population and which tends to be overlooked,^28^ a view born out to a degree by Allen’s^29^ work on commissioning processes for district nursing. Using the lens of workforce development, it is possible to theorize that a multiplicity of factors described above have the potential to inhibit the growth, capacity and capability of this workforce. This study provides an explanatory framework for the apparent paradox between a policy environment that implies growth and development in the district nursing workforce against a decline in numbers and morale. Further investigation is required in other settings to test whether this explanatory framework is of value when the financing mechanism is different.

This is a qualitative study in one setting with only senior nurses as participants and therefore has limitations in that it can be generalized only at the theoretical level. New insights have been offered through an explanatory framework, based on workforce development theory. The involvement of one researcher in this study may be seen as a limitation, but this was mitigated by using analysis verification techniques in the interviews. The use of a separate expert group to test the analysis for further insights or contradictory views assisted in ensuring trustworthiness and credibility of the findings.

## Conclusion

There is an apparent paradox between health policies which promote more care within and closer to home and the reported decline in district nursing services. Using the lens of workforce development theory, a multiplicity of factors with potentially inhibiting influences on growth and development have been identified. An explanatory framework has been offered that includes the nature of the nursing labour market, human resource practices, career advancement opportunities as well as the contractual context and the economic environment. The interlinking network of factors requires attention from policy actors in provider and commissioner organizations. The extent to which this framework is valid in other countries with different financing mechanisms requires further investigation.
